# Neutrophil extracellular traps promote erectile dysfunction in rats with diabetes mellitus by enhancing NLRP3-mediated pyroptosis

**DOI:** 10.1038/s41598-024-67281-6

**Published:** 2024-07-16

**Authors:** Ying Xu, Yan Ren, Wenli Zou, Shuiyu Ji, Wei Shen

**Affiliations:** 1 Department of Urology, Urology and Nephrology Center, Zhejiang Provincial People’s Hospital (Affiliated People’s Hospital), Hangzhou Medical College, Hangzhou, Zhejiang China; 2 Department of Nephrology, Urology and Nephrology Center, Zhejiang Provincial People’s Hospital (Affiliated People’s Hospital), Hangzhou Medical College, No. 158 Shangtang Road, Hangzhou, 310014 Zhejiang China

**Keywords:** Cell biology, Pathogenesis

## Abstract

Erectile dysfunction (ED) is the most prevalent consequences in men with diabetes mellitus (DM). Recent studies demonstrates that neutrophil extracellular traps (NETs) play important roles in DM and its complications. Nevertheless, whether NETs are involved in ED remains unknown. This work intended to explore the role and mechanisms of NETs in ED in the context of DM. Here, we observed that NET generation and pyroptosis were promoted in DM rats with ED compared with controls. Mechanistically, NETs facilitated NLRP3 inflammasome activation and subsequently triggered pyroptosis under high glucose stress, ultimately leading to ED. Intriguingly, DNase I (a NET degrading agent) alleviated ED and corpus cavernosum injury in DM rats. Overall, NETs might induce ED in DM by promoting NLRP3-mediated pyroptosis in the corpus cavernosum.

## Introduction

Erectile dysfunction (ED) is a prevalent male disorder, referring to the incapability to attain or sustain a penile erection adequate for satisfactory sexual intercourse^1,2^. ED has resulted in a diminished quality of life and decreased economic output among males, posing a significant economic strain on the community^3^. Diabetes is the second most frequent cause of ED, and approximately 50% to 75% of diabetic patients suffer from ED^4^. Worryingly, the incidence of diabetic ED continues to rise rapidly and there remains a lack of effective therapeutic approaches^4^. Therefore, it is imperative to explore the underlying mechanisms of ED in the context of DM to develop effective interventions.

Within the circulation system, neutrophils act as the primary immune cells implicated in inflammatory responses^5^. In addition to the ability to secrete antimicrobial compounds and perform phagocytic killing, neutrophils can also eliminate pathogens via releasing the structures known as neutrophil extracellular traps (NETs)^6^. NETs are web-shaped formations comprising relaxed nuclear chromatin and granular proteins, such as neutrophil elastase (NE), cathepsin G, and myeloperoxidase (MPO)^7^. Recent research has highlighted the implications of NETs for the pathogenesis of DM and its several complications, such as impaired wound healing, stroke and retinopathy^8,9^. However, whether NETs are implicated in the pathogenesis of ED are still unknown.

Accumulating evidence indicates that NETs interact with the NOD-like receptor family, pyrin domain-containing protein 3 (NLRP3) inflammasome to aggravate inflammatory responses^10–12^. NLRP3-mediated pyroptosis is demonstrated as the core pathogenic mechanism of diabetic ED^13^. Pyroptosis, an emerging programmed cell death type, is facilitated by multiple cytosolic pattern recognition receptors, with NLRP3 being the most extensively studied receptor^14,15^. Besides NLRP3, apoptosis-associated speck-like protein containing a CARD (ASC), caspase-1, gasdermin D (GSDMD), and interleukin-1β (IL-1β) are the essential molecules in this process^15^. Commonly, NLRP3 assembles with ASC and pro-caspase-1 upon activation, forming NLRP3 inflammasome, subsequently triggering the cleavage of caspase-1^16^. The activated caspase-1 then cleaves pro-IL-1β and pro-IL-18, inducing the generation of mature IL-1β and IL-18^17^. Additionally, caspase-1 cleaves GSDMD, which conduces to N-terminal fragment (GSDMD-N) release^18^. GSDMD-N facilitates cell membrane pore formation, leading to distinct morphological alterations, such as cytoplasm swelling, membrane fracture, and inflammatory factor release, which promotes inflammatory responses^18,19^. Despite that pyroptosis is reportedly the key pathogenic mechanisms of ED under different pathological conditions^15,20,21^, little is known about its molecular mechanisms in this disorder, especially in the context of DM, and further investigation is warranted.

Herein, both cell and animal experiments were carried out to figure out the role of NETs in erectile function in DM. Besides, we attempted to prove that NLRP3-mediated pyroptosis is implicated in diabetic ED pathogenesis. This study provides novel ideas for the mechanisms of diabetic ED and furnishes a direction for follow-up studies, which would benefit the development of effective therapeutic strategies for this disease.

## Results

### NET generation was increased in diabetic ED rats

**Figure 1:**
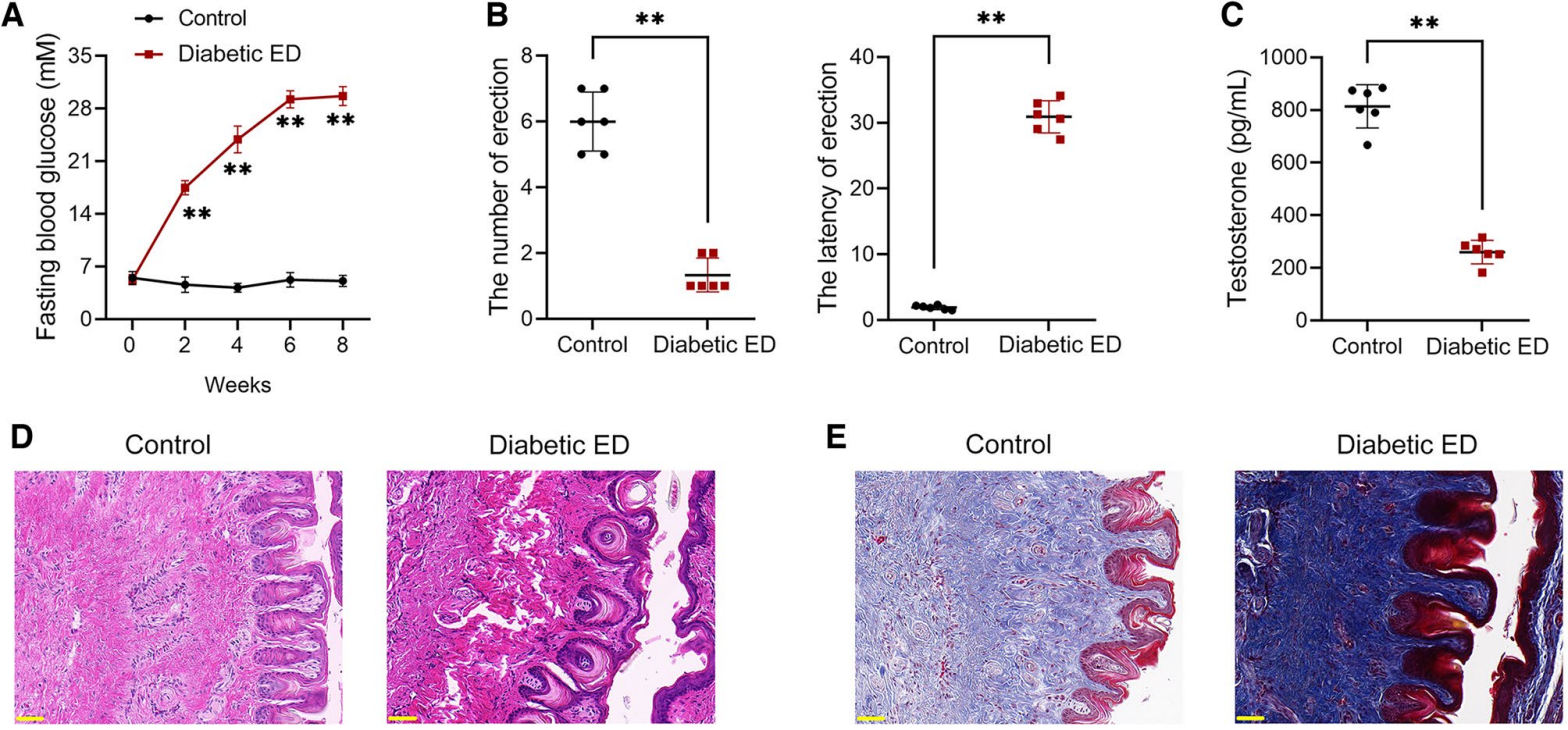
A rat model of diabetic ED was successfully constructed. (**A**) Assessment of the fasting blood glucose level in rats (n = 6/group). (**B**) Assessment of erection number and latency in rats (n = 6/group). (**C**) Assessment of the serum testosterone level using ELISA (n = 6/group). (**D)** Histopathological examination of the corpus cavernosum tissues of rats using HE staining (n = 6/group; scale bar = 50 μm). (**E**) Histopathological examination of the corpus cavernosum tissues of rats using Masson staining (n = 6/group; scale bar = 50 μm). Data were expressed as mean ± standard deviation. **p < 0.01. *ED* erectile dysfunction, *HE* hematoxylin–eosin, *ELISA* enzyme-linked immunosorbent assay.

To explore whether NET dysregulation is implicated in diabetic ED pathogenesis, we established a diabetic ED rat model using streptozotocin (STZ) treatment and apomorphine (APO) testing. Here, the fasting blood glucose was significantly elevated in diabetic ED rats relative to controls (*p* < 0.01; Fig. 1A). The erectile function of rats in each group was examined. Diabetic ED rats were found to have a markedly smaller numbers of erections and a longer erection latency than controls (*p* < 0.01; Fig. 1B). Besides, the serum testosterone level of diabetic ED rats was markedly reduced relative to that of controls (*p* < 0.01; Fig. 1C). Through HE staining, we observed endothelial cell degeneration in the corpus cavernosum tissues of diabetic ED rats, including vascular congestion, diffuse sinusoidal injury, vascular wall thickening, and interstitial fibrosis, relative to controls (Fig. 1D). Besides, Masson staining revealed the markedly elevated collagen content in the corpus cavernosum tissues of diabetic ED rats relative to that in controls (Fig. 1E). These results suggested the successful construction of the diabetic ED rat model.

**Figure 2:**
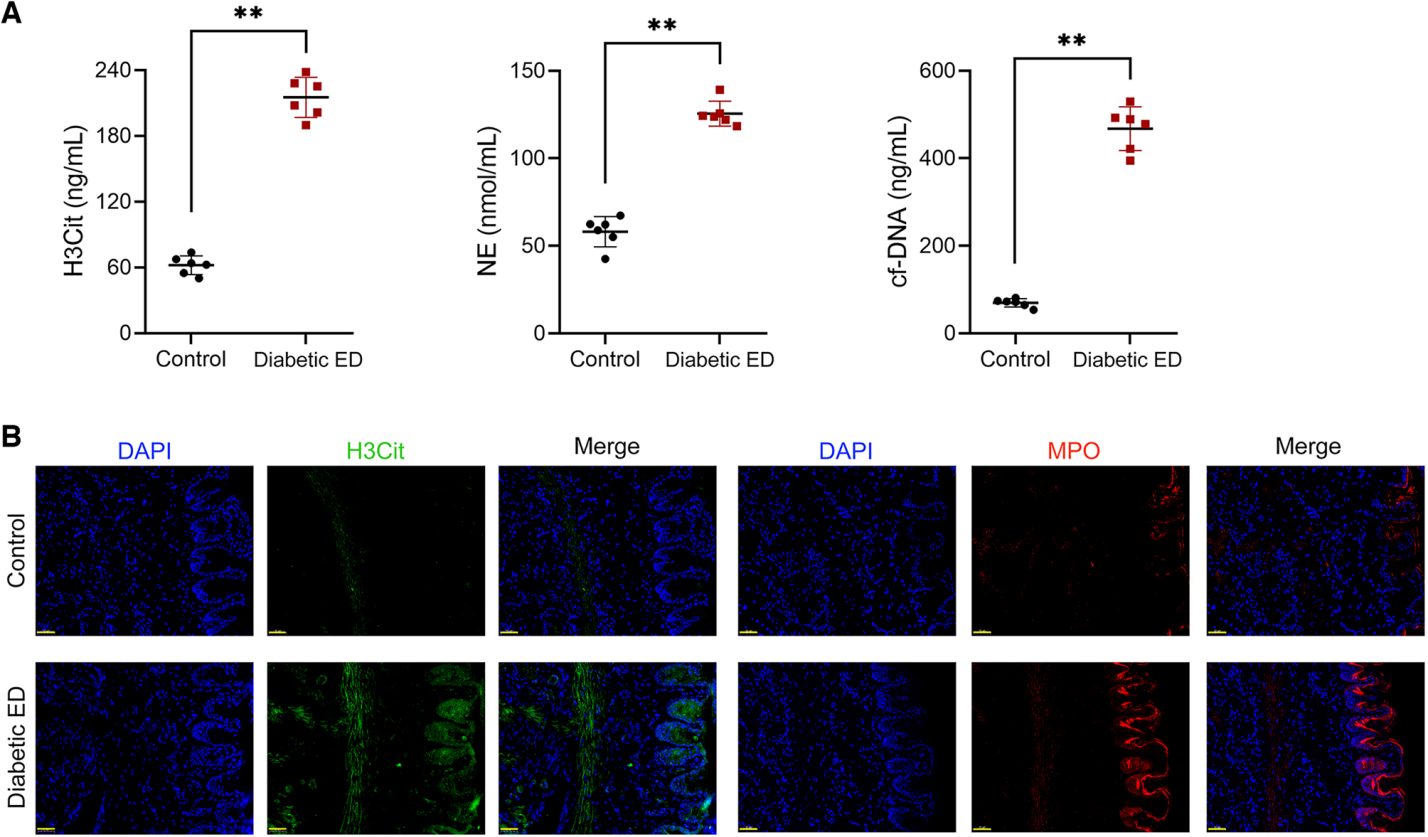
NET formation was elevated in diabetic ED rats. (**A**) Assessment of the levels of plasma H3Cit, NE, and cf-DNA (n = 6/group). (**B**) Assessment of expression levels of H3Cit and MPO in the corpus cavernosum tissues of rats using immunofluorescence (n = 6/group; scale bar = 50 μm). Data were expressed as mean ± standard deviation. **p < 0.01. *NET* neutrophil extracellular trap, *ED* erectile dysfunction, *H3Cit* citrullinated histone H3, *NE* neutrophil elastase, *cf-DNA* cell-free DNA, *MPO* myeloperoxidase.

Subsequently, the levels of NET markers (H3Cit, NE, cf-DNA, and MPO) were detected in rats in each group. Enzyme-linked immunosorbent assay (ELISA) revealed that the levels of plasma H3Cit, NE, and cell-free DNA (cf-DNA) were markedly upregulated in diabetic ED rats relative to those in controls (*p* < 0.01; Fig. 2A). Additionally, immunofluorescence showed that H3Cit and MPO expression were markedly upregulated in the corpus cavernosum tissues of diabetic ED rats compared with those in controls (Fig. 2B).

### NLRP3-mediated pyroptosis was promoted in the *corpus* cavernosum tissues of diabetic ED rats

**Figure 3:**
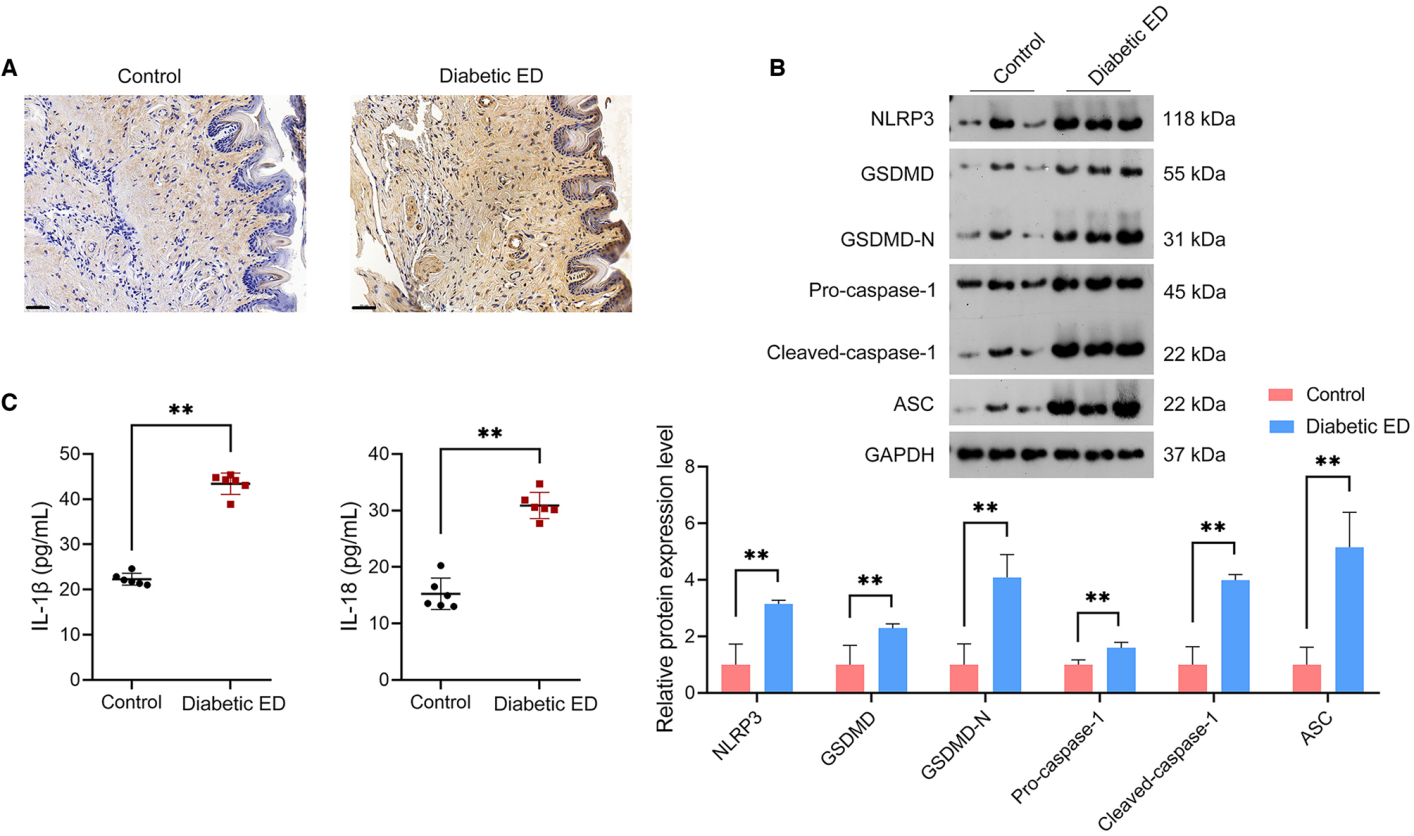
NLRP3 expression and pyroptosis were enhanced in the corpus cavernosum tissues of diabetic ED rats. (**A**) Assessment of NLRP3 expression in the corpus cavernosum tissues of rats using immunohistochemistry (n = 6/group; scale bar = 50 μm). (**B**) Assessment of the protein expression levels of NLRP3, GSDMD, GSDMD-N, pro-caspase-1, cleaved-caspase-1, and ASC in the corpus cavernosum tissues of rats using western blotting (n = 6/group); the uncropped protein blots can be seen in Supplementary file 1. (**C**) Assessment of plasma IL-1β and IL-18 levels in rats using ELISA (n = 6/group). Data were expressed as mean ± standard deviation. **p < 0.01. *NLRP3* NOD-like receptor family, pyrin domain-containing protein 3, *ED* erectile dysfunction, *GSDMD* gasdermin D, *GSDMD-N* N-terminal proteolytic fragment of gasdermin D, *ASC* apoptosis-associated speck-like protein containing a CARD, *IL* interleukin, *ELISA* enzyme-linked immunosorbent assay.

NLRP3 expression in the corpus cavernosum tissues of rats was detected firstly to determine the involvement of NLRP3 in diabetic ED development. Immunohistochemistry showed an elevation of NLRP3 expression in the corpus cavernosum tissues of diabetic ED rats relative to that in controls (Fig. 3A). Accordingly, we hypothesized that NLRP3 dysregulation might affect pyroptosis in diabetic ED and evaluated pyroptosis markers (GSDMD, GSDMD-N, pro-caspase-1, cleaved-caspase-1, ASC, IL-1β, and IL-18). Western blotting revealed that NLRP3, GSDMD, GSDMD-N, pro-caspase-1, cleaved-caspase-1, and ASC protein expression levels were significantly augmented in the corpus cavernosum tissues of diabetic ED rats relative to those in controls (*p* < 0.01; Fig. 3B). Moreover, ELISA showed markedly higher plasma IL-1β and IL-18 levels in diabetic ED rats than those in controls (*p* < 0.01; Fig. 3C).

### NET degradation alleviated diabetic ED symptoms and inhibited pyroptosis in *corpus* cavernosum tissues

**Figure 4:**
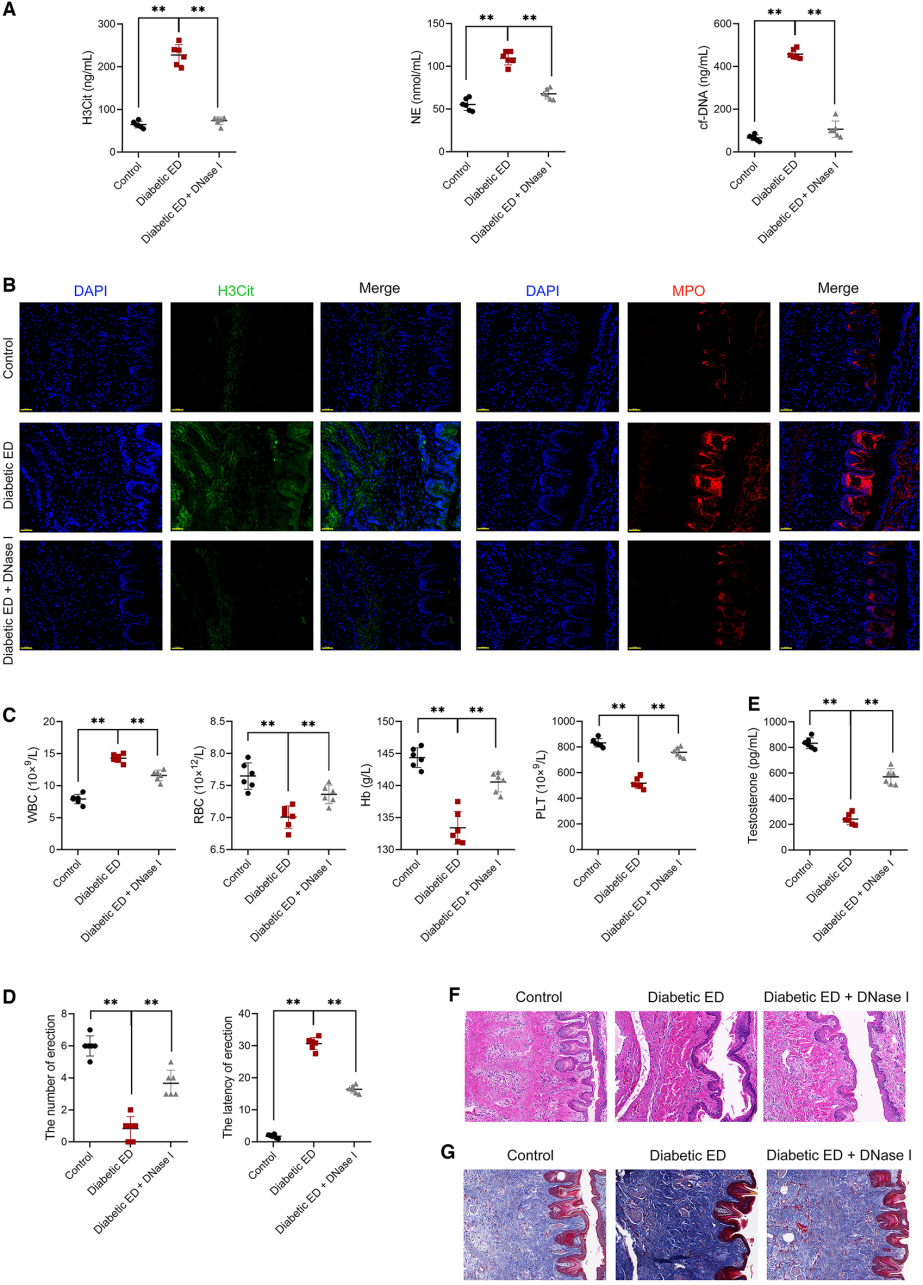
NET degradation alleviated diabetic ED symptoms of rats. (**A**) Assessment of the levels of plasma H3Cit, NE, and cf-DNA (n = 6/group). (**B**) Assessment of expression levels of H3Cit and MPO in the corpus cavernosum tissues of rats using immunofluorescence (n = 6/group; scale bar = 50 μm). (**C**) Blood routine examination of WBC, RBC, Hb, and PLT levels in rats (n = 6/group). (**D**) Assessment of erection number and latency in rats (n = 6/group). (**E**) Assessment of the serum testosterone level using ELISA (n = 6/group). (**F)** Histopathological examination of the corpus cavernosum tissues of rats using HE staining (n = 6/group; scale bar = 50 μm). (**G**) Histopathological examination of the corpus cavernosum tissues of rats using Masson staining (n = 6/group; scale bar = 50 μm). Rats in diabetic ED + DNase I group were intraperitoneally injected with DNase I (a NET degrading agent) at the dose of 1 mg/kg for 8 successive days. Meanwhile, rats in Control and diabetic ED groups were intraperitoneally injected with the same amount of normal saline. Data were presented as mean ± standard deviation. **p < 0.01. *NET* neutrophil extracellular trap, *ED* erectile dysfunction, *H3Cit* citrullinated histone H3, *NE* neutrophil elastase, *cf-DNA* cell-free DNA, *MPO* myeloperoxidase, *WBC* white blood cell, *RBC* red blood cell, *Hb* hemoglobin, *PLT* platelet, *ELISA* enzyme-linked immunosorbent assay, *HE* hematoxylin–eosin.

**Figure 5:**
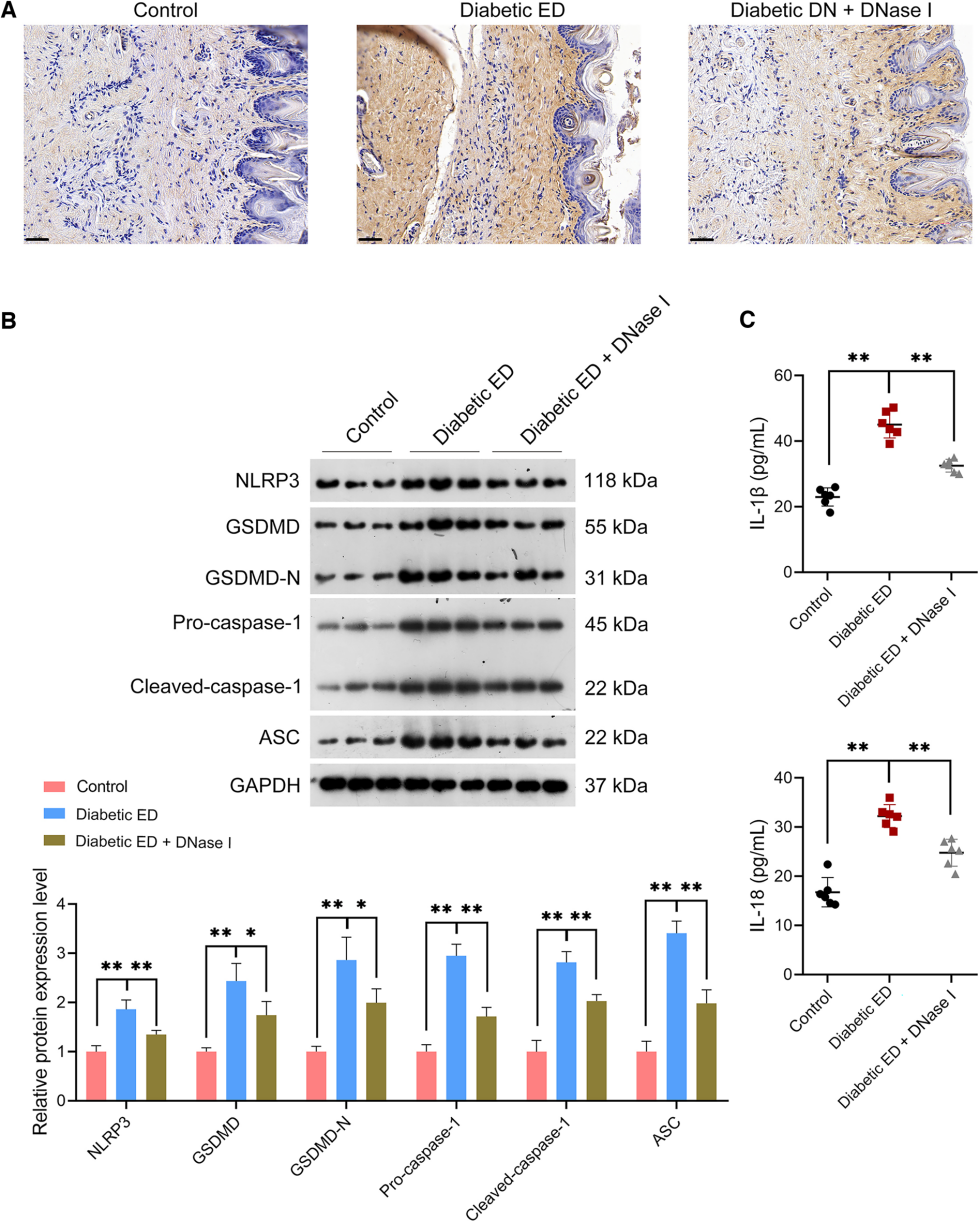
NET degradation inhibited the pyroptosis in the corpus cavernosum tissues of diabetic ED rats. (**A**) Assessment of NLRP3 expression in the corpus cavernosum tissues of rats using immunohistochemistry (n = 6/group; scale bar = 50 μm). (**B**) Assessment of the protein expression levels of NLRP3, GSDMD, GSDMD-N, pro-caspase-1, cleaved-caspase-1, and ASC in the corpus cavernosum tissues of rats using western blotting (n = 6/group); the uncropped protein blots can be seen in Supplementary file 2. (**C**) Assessment of plasma IL-1β and IL-18 levels in rats using ELISA (n = 6/group). Rats in diabetic ED + DNase I group were intraperitoneally injected with DNase I (a NET degrading agent) at the dose of 1 mg/kg for 8 successive days. Meanwhile, rats in Control and diabetic ED groups were intraperitoneally injected with the same amount of normal saline. Data were expressed as mean ± standard deviation. **p < 0.01. *NET* neutrophil extracellular trap, *NLRP3* NOD-like receptor family, pyrin domain-containing protein 3, *GSDMD* gasdermin D, *GSDMD-N* N-terminal proteolytic fragment of gasdermin D, *ASC* apoptosis-associated speck-like protein containing a CARD, *IL* interleukin, *ELISA* enzyme-linked immunosorbent assay.

To further determine the promotive function of NETs in NLRP3-mediated pyroptosis in diabetic ED, diabetic ED rats were intraperitoneally injected with the NET degrading agent, DNase I. Here, DNase I treatment markedly reduced NET generation in diabetic ED rats (Fig. 4A, B). Through blood routine examination, the white blood cells (WBC) level in diabetic ED rats was significantly higher than that in controls, while the red blood cells (RBC), hemoglobin (Hb), and platelets (PLT) levels were lower, which was reversed following DNase I treatment (*p* < 0.01; Fig. 4C). Notably, ED (*p* < 0.01; Fig. 4D, E) and histopathological injury (Fig. 4F, G) of diabetic ED rats were effectively mitigated following DNase I treatment. Furthermore, DNase I treatment reduced NLRP3, GSDMD, GSDMD-N, pro-caspase-1, cleaved-caspase-1, ASC, IL-1β, and IL-18 expression in the corpus cavernosum tissues of diabetic ED rats (Fig. 5A–C).

### NETs facilitated NLRP3-mediated pyroptosis of high glucose (HG)-treated rat *corpus* cavernosum smooth muscle cells (CCSMCs)

**Figure 6:**
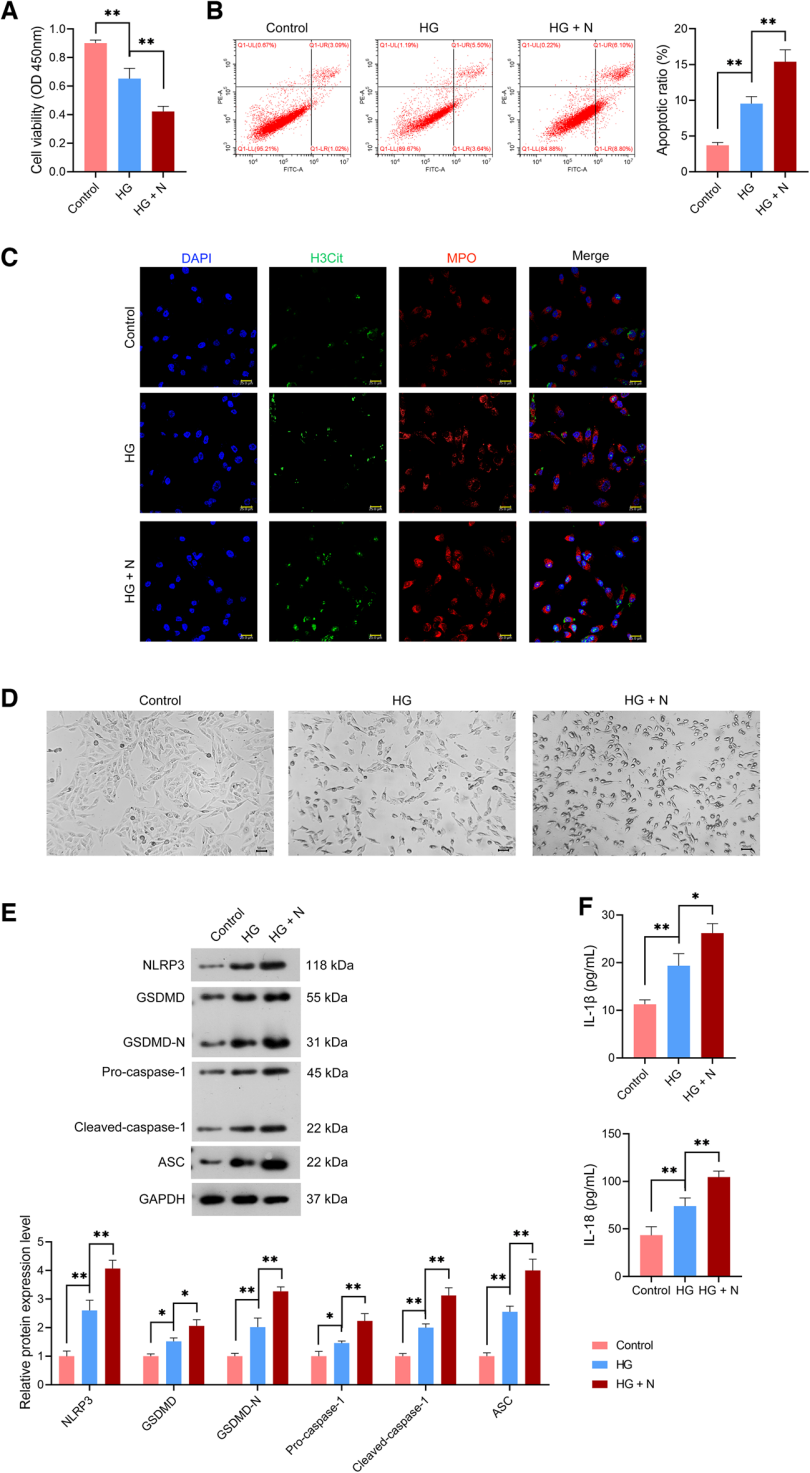
NETs amplified the pyroptosis of HG-treated rat CCSMCs. (**A**,**B**) Assessment of the viability and apoptosis in rat CCSMCs using CCK-8 assay and flow cytometry, respectively. (**C**) Assessment of the expression levels of H3Cit and MPO in CCSMCs using immunofluorescence (scale bar = 25 μm). (**D**) Observation of CCSMC pyroptosis under a microscope (scale bar = 50 μm). (**E**) Assessment of the protein expression levels of NLRP3, GSDMD, GSDMD-N, pro-caspase-1, cleaved-caspase-1, and ASC in CCSMCs using western blotting; the uncropped protein blots can be seen in Supplementary file 3. (**F**) Assessment of IL-1β and IL-18 levels in CCSMCs using ELISA. CCSMCs were subjected to HG (25 mM) treatment or/and co-cultured with 1 × 10^4^ neutrophils (N) for 24 h. CCSMCs cultured in 5 nM glucose served as controls. Data were presented as mean ± standard deviation. *p < 0.05 and **p < 0.01. *NET* neutrophil extracellular trap, *HG* high glucose, *CCSMCs* corpus cavernosum smooth muscle cells, *CCK-8* cell counting kit-8, *H3Cit* citrullinated histone H3, *MPO* myeloperoxidase, *NLRP3* NOD-like receptor family, pyrin domain-containing protein 3, *GSDMD* gasdermin D, *GSDMD-N* N-terminal proteolytic fragment of gasdermin D, *ASC* apoptosis-associated speck-like protein containing a CARD, *IL* interleukin, *ELISA* enzyme-linked immunosorbent assay.

We further performed in vitro experiments to confirm our hypothesis by treating rat CCSMCs with HG and co-culturing them with or without rat neutrophils (N). Here, the cell viability of HG-treated CCSMCs was markedly reduced relative to that of controls, and the decreased viability was aggravated when co-cultured with neutrophils (*p* < 0.01; Fig. 6A). Moreover, HG treatment significantly promoted CCSMC apoptosis, which was further enhanced when CCSMCs were co-cultured with neutrophils (*p* < 0.01; Fig. 6B).

In terms of NET generation, H3Cit and MPO expression were elevated in HG-induced CCSMCs relative to those in controls, which were further upregulated when CCSMCs were exposed to neutrophils (Fig. 6C). Notably, we observed that HG-induced CCSMCs displayed pyroptotic-like morphological characteristics and membrane ballooning relative to controls, especially when co-cultured with neutrophils (Fig. 6D). Furthermore, western blotting revealed notable increases NLRP3, GSDMD, GSDMD-N, pro-caspase-1, cleaved-caspase-1, and ASC protein expression in HG-treated CCSMCs, especially when co-cultured with neutrophils (*p* < 0.05; Fig. 6E). ELISA showed IL-1β and IL-18 levels were markedly higher in HG-treated CCSMCs than those in controls, which were further elevated with neutrophils co-cultured (*p* < 0.05; Fig. 6F).

**Figure 7:**
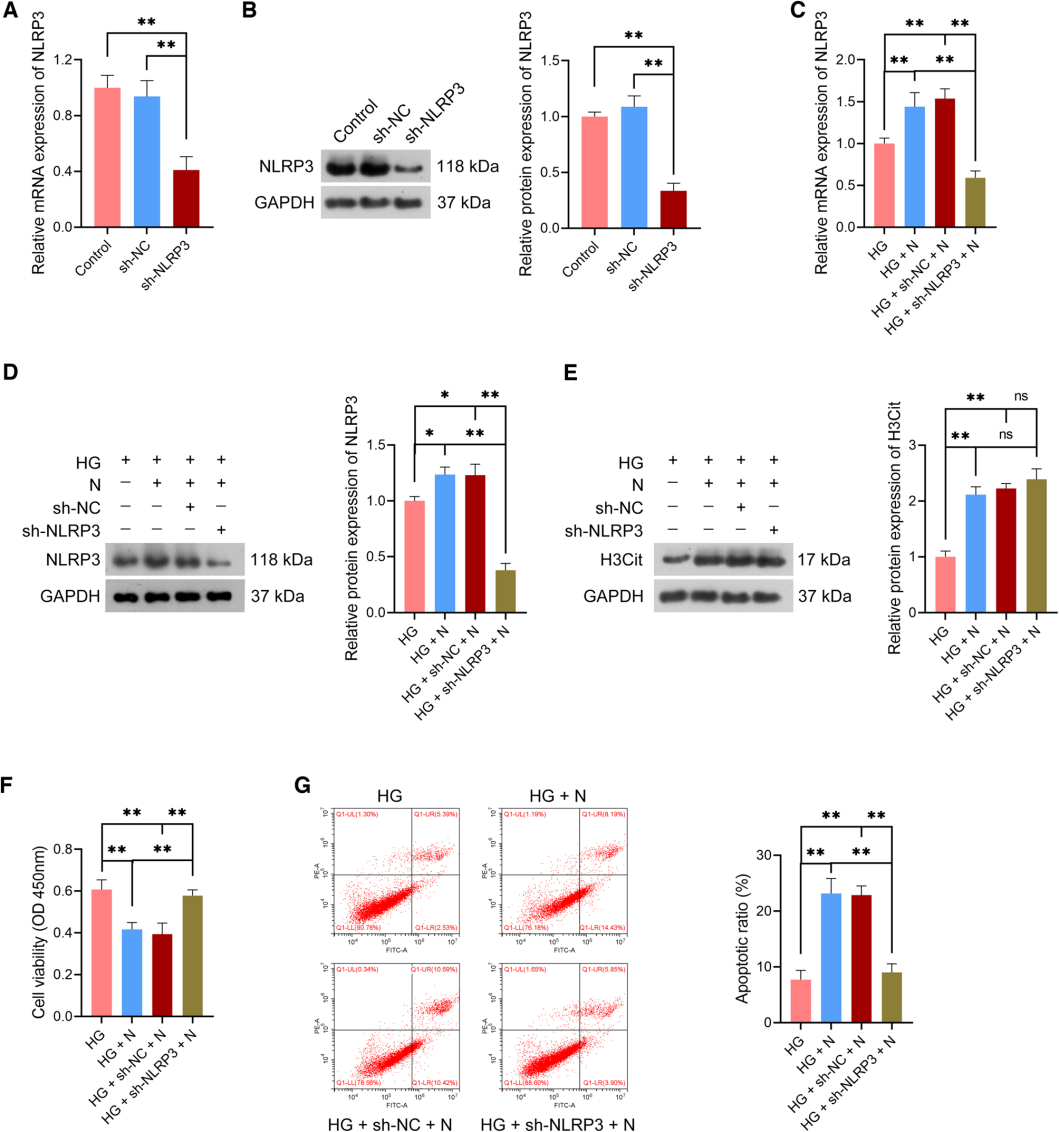
NETs inhibited the proliferation of HG-treated rat CCSMCs by NLRP3 activation. (**A**,**B**) Evaluation of sh-NLRP3 transfection efficiency in CCSMCs using qRT-PCR and western blotting; the uncropped protein blots can be seen in Supplementary file 4. (**C**,**D**) Assessment of NLRP3 expression in HG-treated CCSMCs using qRT-PCR and western blotting; the uncropped protein blots can be seen in Supplementary file 4. (**E**) Assessment of H3Cit expression in HG-treated CCSMCs using western blotting; the uncropped protein blots can be seen in Supplementary file 4. (**F**,**G**) Assessment of the viability and apoptosis in HG-treated CCSMCs using CCK-8 assay and flow cytometry, respectively. (**C**–**G**) sh-NC- or sh-NLRP3-transfected CCSMCs were subjected to HG (25 mM) treatment or/and co-cultured with 1 × 10^4^ neutrophils (N) for 24 h. CCSMCs cultured in 5 nM glucose served as controls. Data were presented as mean ± standard deviation. *p < 0.05 and **p < 0.01. *NET* neutrophil extracellular trap, *NLRP3* NOD-like receptor family, pyrin domain-containing protein 3, *HG* high glucose, *CCSMCs* corpus cavernosum smooth muscle cells, *qRT-PCR* quantitative real-time polymerase chain reaction, *H3Cit* citrullinated histone H3, *CCK-8* cell counting kit-8.

To ascertain whether NETs enhance rat CCSMC pyroptosis through the NLRP3 axis, empty vector and short hairpin RNA targeting NLRP3 (sh-NLRP3) lentiviruses were respectively transfected to rat CCSMCs. We observed that NLRP3 expression was markedly downregulated in sh-NLRP3-transfected CCSMCs relative to sh-NC-transfected CCSMCs (*p* < 0.01; Fig. 7A, B). These results indicated the successful establishment of NLRP3-deficient CCSMCs.

**Figure 8:**
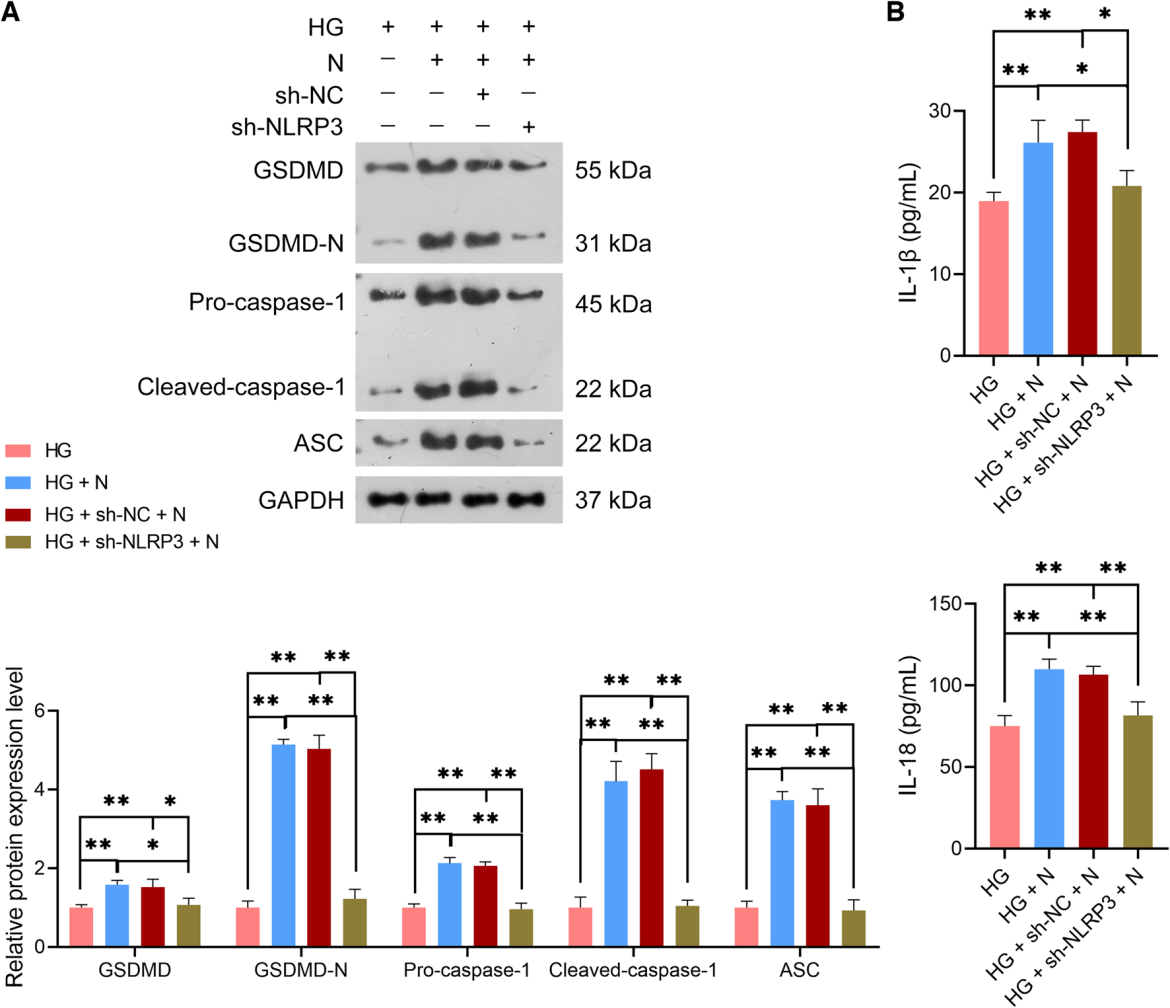
NETs promoted NLRP3-mediated pyroptosis of HG-treated rat CCSMCs. (**A**) Assessment of the protein expression levels of NLRP3, GSDMD, GSDMD-N, pro-caspase-1, cleaved-caspase-1, and ASC in HG-treated CCSMCs using western blotting; the uncropped protein blots can be seen in Supplementary file 5. (**B**) Assessment of IL-1β and IL-18 levels in HG-treated CCSMCs using ELISA. sh-NC- or sh-NLRP3-transfected CCSMCs were subjected to HG (25 mM) treatment or/and co-cultured with 1 × 10^4^ neutrophils (N) for 24 h. CCSMCs cultured in 5 nM glucose served as controls. Data were presented as mean ± standard deviation. *p < 0.05, **p < 0.01, and ns denotes no significant difference between groups. *NET* neutrophil extracellular trap, *NLRP3* NOD-like receptor family, pyrin domain-containing protein 3, *HG* high glucose, *CCSMC* corpus cavernosum smooth muscle cell, *GSDMD* gasdermin D, *GSDMD-N* N-terminal proteolytic fragment of gasdermin D, *ASC* apoptosis-associated speck-like protein containing a CARD, *IL* interleukin, *ELISA* enzyme-linked immunosorbent assay.

Subsequently, sh-NC- and sh-NLRP3-transfected CCSMCs were treated with HG and co-cultured with neutrophils for functional assays. Here, sh-NLRP3 abolished the promotive effects of neutrophils on NLRP3 expression in HG-treated CCSMCs (*p* < 0.01; Fig. 7C, D). However, sh-NLRP3 did not affect H3Cit protein expression level in HG-treated CCSMCs (*p* > 0.05; Fig. 7E). Moreover, sh-NLRP3 was observed to eliminate the effects of neutrophils on suppressing the viability and promoting apoptosis of HG-treated CCSMCs compared with sh-NC (*p* < 0.01; Fig. 7F, G). Furthermore, sh-NLRP3 undid the promotive role of neutrophils on HG-treated CCSMC pyroptosis relative to sh-NC, as GSDMD, GSDMD-N, pro-caspase-1, cleaved-caspase-1, ASC, IL-1β, and IL-18 expression were downregulated (*p* < 0.01; Fig. 8A, B).

## Discussion

NETs contribute to the pathogenesis of diabetes and its complications^11,22,23^. Nevertheless, whether NETs are involved in diabetic ED is still unknown. The current research fills this void in understanding. Herein, we constructed a DM rat model induced by STZ, and rats without any erection after APO testing were thought to suffer from ED. As a result, NET generation was found to be elevated in diabetic ED rats, which was associated with ED. Mechanistically, it was demonstrated that the pathogenic role of NETs in diabetic ED might be partly owing to the promotion of NLRP3-mediated pyroptosis. Our findings emphasize a new mechanism of ED in the context of DM, which exacerbates corpus cavernosum injury.

NETs are frequently characterized as a crucial part of the anti-bacteria process; nevertheless, due to the non-specific nature of NET components, an overabundance of NETs can often lead to inflammation and tissue lesions^24^. Chronic inflammation is a vital part of ED pathogenesis and a possible middle phase of endothelial dysfunction, particularly in metabolic disorders, including diabetes^25^. At the beginning of this study, the NET formation markers (H3Cit, NE, cf-DNA, and MPO) were observed to be upregulated in diabetic ED rats compared with those in controls. In line with our findings, previous research revealed increased NET generation in DN^26,27^. Thus, we hypothesized a significant role of NETs in diabetic ED pathogenesis and subsequently explored related mechanisms.

Increasing studies reveal NETs can interact with NLRP3 inflammasome to aggravate inflammation in various diseases^10,11,28^. Herein, we found that NLRP3 expression was higher in diabetic ED rats than that in controls, which conformed to the previous investigations into diabetic ED^13^. Notably, neutrophils, the source of NETs, further enhanced NLRP3 expression in rat CCSMCs with decreased cell viability and enhanced apoptosis, under the condition of HG. This suggested that NETs might promote diabetic ED progression by activating NLRP3 signaling. The NLRP3 inflammasome is known to recognize internal danger signals and then activate caspase-1 and IL-1β, ultimately triggering inflammatory responses^29^. Additionally, NLRP3 activation conduces to caspase-1 maturation, facilitating IL-1β and IL-18 secretion, thereby triggering pyroptosis^30^. Accumulating evidence indicates that NLRP3 activation contributes to the development and progression of ED^20,31^. Inhibition of NLRP3-mediated pyroptosis of nerve is associated with ameliorated ED^20^. On the other hand, prior data demonstrated that the promotion of pyroptosis in corpus cavernosum could conduce to ED in rats^15,32^. Based on these facts, we explored the effects of NETs on pyroptosis in diabetic ED.

Unlike apoptosis, pyroptosis is an inflammatory caspase-dependent form of programmed cell death, marked by cell membrane pore formation, membrane rupture, cell swelling, and cell content release^33^. Central to pyroptosis is the activation of the inflammasome, especially the NLRP3 inflammasome, which is among the most extensively researched^34^. Upon detecting danger or pathogen-associated molecular patterns, NLRP3 recruits pro-caspase-1 via ASC to assemble the NLRP3 inflammasome^16^. This assembly prompts the transformation of pro-caspase-1 into its active state, subsequently processing pro-IL-1β and pro-IL-18 into their mature forms^17^. GSDMD acts as the primary executor of pyroptosis. GSDMD is cleaved by caspase-1 into GSDMD-N, which creates pores in the cellular membrane^35^. This process ultimately induces cell death and facilitates the release of pro-inflammatory factors like IL-1β and IL-18, igniting a potent inflammatory reaction^35^. Herein, GSDMD, GSDMD-N, pro-caspase-1, c-caspase-1, ASC, IL-1β, and IL-18 levels were elevated in the corpus cavernosum tissues of diabetic ED rats relative to controls, indicating the promotion of pyroptosis in diabetic ED. These data were in line with the previous findings that pyroptosis was promoted in diabetic ED^13^. Significantly, neutrophils aggravated the pyroptosis of rat CCSMCs, under the condition of HG. However, NLRP3 knockdown abolished the effects of NETs on decreasing the viability and enhancing the apoptosis of HG-treated rat CCSMCs. Furthermore, NLRP3 downregulation eliminated the promoting function of NETs on the pyroptosis of HG-treated rat CCSMCs. A prior investigation demonstrated that NLRP3 reduction could inhibit the pyroptosis in corpus cavernosum tissues, thus improving the erectile function of a rat model of diabetic ED^13^. Collectively, our data imply that NETs might act as pathogenic factors in diabetic ED by promoting NLRP3-mediated pyroptosis in the corpus cavernosum.

Finally, to further ascertain the pathogenic role of NETs in diabetic ED, we conducted in vivo experiment by treating diabetic ED rats with DNase I, a degrading agent of NETs. DNase I treatment rescued blood routine abnormities and alleviated histopathological injury of diabetic ED rats. Previous research suggested that inhibition of NLRP3-mediated pyroptosis could alleviate ED in diabetic rats^13^. Nevertheless, there is little report of the involvement of NETs in the pyroptosis mechanism in ED. Here, we for the first time observed that NET degradation improved the erectile function of diabetic ED rats and inhibited NLRP3-mediated pyroptosis in the rat cavernosum tissues. Collectively, these findings indicate that NETs might induce ED in DM by promoting NLRP3-mediated pyroptosis.

This study has some limitations. Firstly, while the study verified the relationship between NETs and NLRP3 in diabetic ED in vivo and in vitro, the signaling pathway by which NETs induce NLRP3-mediated pyroptosis (such as the NF-κB pathway) is yet to be explored. Besides, other inflammasomes, such as AIM2 inflammasome^36,37^, pyrin inflammasome^38^ are also involved in NETs-driven inflammation. Further experiments should be conducted to reveal whether other inflammasomes functions in the roles of NETs in diabetic ED. Secondly, we induced the type 1 DM rat models, and the relative mechanism of ED was investigated in the context of type 1 DM. It is still unclear whether such phenomenon is appropriate to elucidate the mechanism of ED in the context of type 2 DM, and therefore, further investigations are needed. Thirdly, the use of animal models to simulate diabetic ED might not fully capture the complexity and diversity of human diseases. Therefore, future studies should incorporate in-depth exploration of the specific molecular pathways involved in NETs-mediated pyroptosis and identification of downstream molecules. Besides, additional studies are required to assess the efficacy of NET-, NLRP3-, and pyroptosis-targeted strategies for the treatment of ED in DM patients, which is essential for the translation of these findings into clinically therapeutic approaches.

## Conclusions

This study revealed that NETs could induce ED in DM, which might be partly due to their promotive role in NLRP3-mediated pyroptosis. In general, our findings suggest that intervention strategies targeting NETs are expected to be potential treatments for diabetic ED. This work furthers the understanding of diabetic ED pathogenesis and furnishes research directions for diabetic ED treatment.

## Methods

### Experimental animals

Thirty-five specific pathogen-free male Wistar rats (8 weeks old; 240 g), supplied by the Institute of Comparative Medicine, Yangzhou University (Yangzhou, China), were used for research. All rats were bred under the following conditions: a 12-h light/dark cycle; 19–25 °C; 35–55% humidity, and provided with ad libitum food and water. The animal experiments were permitted by the Experimental Animal Ethics Committee of Yangzhou University (No. 202304016), which conformed to the National Institutes of Health Guidelines for the Care and Use of Laboratory Animals, and methods were reported in accordance with ARRIVE guidelines.

### Animal modeling and treatments

After 7-day adaptive feeding, 12 rats were randomly selected to induce a type 1 DM model. According to the previous research^39,40^, the 12 rats were intraperitoneally injected with STZ (60 mg/kg; #S0130, Sigma, USA), dissolved in cold citrate buffer (pH 4.5; 0.1 M). Moreover, six normal rats were used as controls (control group), which were treated with the equal volume of odium citrate buffer via intraperitoneal injection. One day later, the blood glucose of rats was monitored, and those presenting blood glucose exceeding 16.7 mM for three successive days were perceived to be type 1 diabetic^41^. After 8 weeks, diabetic rats were further screened for ED using APO (Sigma-Aldrich, St. Louis, MO, USA). Following spending 10 min in a dim, tranquil environment, the rats were subjected to a subcutaneous injection of APO (100 µg/kg) in the nape of their necks. Subsequent behavior was recorded over a 30-min span using a video camera (Nikon, Tokyo, Japan; D7500). An erection was characterized by noticeable penile engorgement or the exposure of the glans. Diabetic rats that did not exhibit any erection were thought to undergo ED (diabetic ED) based on the prior study^42^. Finally, 12 diabetic ED rats were successfully determined.

To further confirm the effects of NETs on ED in DM, the 12 diabetic ED rats were assigned to diabetic ED and diabetic ED + DNase I groups (n = 6/group). Rats in diabetic ED + DNase I group were intraperitoneally injected with DNase I (a NET degrading agent; 1 mg/kg; #HY-108882, MedChemExpress, Monmouth Junction, NJ, USA) for 8 successive days to inhibit NET generation. At the same time, control and diabetic ED groups received an intraperitoneal injection with the equivalent amount of normal saline for 8 successive days.

The urine and blood samples were collected, and all rats were euthanatized after anesthetization by 100 mg/kg pentobarbital via intravenous injection^43^ following 8 days of treatment. Then, the kidney and cavernous tissues were collected for later assays.

### Biochemical index and blood routine examination

The rat fasting blood glucose was assessed at weeks 0, 4, 6, and 8 using a glucometer (A36B, Roche, Basel, Switzerland). The rats were moved to metabolic cages one day before euthanasia, 24-h urine and blood were collected. The fresh urine samples were subjected to 15-min centrifugation at 3000 rpm and kept at −20 °C. Then, the rats were injected intraperitoneally with pentobarbital (40 mg/kg) for anesthesia, and the arterial blood (about 5 mL) was drawn from the abdominal aorta. The serum was isolated after centrifugation at 3000 rpm for 5 min and stored at −20 °C. BUN, CREA, and UACR, WBC, RBC, Hb, and PLT were assessed via an Indiko automatic biochemical analyzer (Thermo Scientific, Waltham, MA, USA).

### Neutrophil isolation

Following the supplier's directions, neutrophils were separated from peripheral blood of rats in normal group using a neutrophil isolation kit (#CLS1091, Sangon Biotech, Shanghai, China). Briefly, rat blood was mixed with ethyl starch (1:1) and incubated at 37 °C for 10 min. Then, the supernatant was collected, and the settling red blood cells were retained. The supernatant was mixed with the cell washing solution, then 15-min centrifugation at 500*g*. Subsequently, the supernatant was removed and the settling cells were retained. The cell precipitate was added with the whole blood diluent to make the cell concentration 2 × 10^8^–1 × 10^9^ cells /mL, followed by 20-min centrifugation at 400*g*. A large number of neutrophils were obtained using red blood cell lysate and after removal of red blood cells. The obtained neutrophils were washed and centrifuged at 500*g* for 15 min. After the precipitate was washed twice, the required neutrophils were acquired.

### Cell culture, transfection, and treatment

According to previous research^44^, the CCSMCs were separated from cavernous tissues of rats in normal group and purified using the differential wall adhesion approach. CCSMCs and neutrophils were respectively cultivated in low-glucose DMEM (#C11885500BT, Gibco, Grand Island, NY, USA) comprising fetal bovine serum (FBS; #S9030, Solarbio, Beijing, China) and 1% penicillin–streptomycin (#15070063, Gibco) with 5% CO_2_ at 37 °C.

All plasmids used in this study were synthesized and supplied by GenePharma (Shanghai, China). A sh-NLRP3 was employed for NLRP3 downregulation in CCSMCs. Scrambled shRNA (sh-NC) was used as the control. In brief, CCSMCs were subjected to 72-h transfection after seeding onto six-well plates (2 × 10^5^ cells/well). Transfection experiments were carried out via HighGene transfection kits (#RM09014, ABclonal, Wuhan, China). To evaluate the transfection effectiveness, we applied quantitative real-time polymerase chain reaction (qRT-PCR) and western blotting. The primers of sh-NLRP3 are listed in Table S1.

To ascertain the role of NETs in diabetic ED, CCSMCs were subjected to HG (25 mM) treatment or/and co-cultured with 1 × 10^4^ neutrophils for 24 h. Furthermore, to figure out how NETs affect NLRP3-mediated pyroptosis in CCSMCs, sh-NC- or sh-NLRP3-transfected CCSMCs were exposed to HG and co-cultured with neutrophils. CCSMCs cultured in 5 nM glucose served as controls.

### HE and Masson staining

The kidney or corpus cavernosum tissues from rats were paraffin-embedded, sliced at 5 µm, deparaffinized, and hydrated. Subsequently, the sections were stained with HE (#C0105S, Beyotime, Shanghai, China) or Masson (#G1340, Solarbio, Beijing, China) staining kit following the provided protocols. Finally, the section images were captured by a microscope (CKX53, Olympus, Tokyo, Japan).

### Assessment of erectile function

Rats were housed in a low-light and quiet setting, conducive for observation. Then, the rats received an injection of APO (100 µg/kg) through the neck. Finally, the number of erections and the erection latency within 30 min were tracked and recorded. The erection latency refers to the time span from APO treatment to erection.

### ELISA

ELISA was utilized for the assessment of testosterone, H3Cit, NE, IL-1β, and IL-18 levels in rat plasma or CCSMCs. Optical density (OD) values at 450 nm were assessed by a microplate reader (DR-3518G, Hiwell Diatek, Wuxi, China) and quantified using ImageJ software (NIH, Bethesda, USA). ELISA kits used in this assay were as follows: testosterone (#ml002868, mlbio, Shanghai, China), H3Cit (#YX-22516R, Sinobestbio, Shanghai, China), NE (#ml003280, mlbio), IL-1β (#ml037361, mlbio), and IL-18 (#ml002816, mlbio).

### Detection of cf-DNA

As per the manufacturer’s directions, the cf-DNA level in rat plasma was assessed using a Quant-iTPicoGreen dsDNA kit (#P11496, Thermo Scientific). The cf-DNA level was determined by a microplate reader (DR-3518G, Hiwell Diatek), and the excitation and emission wavelengths were 480 nm and 520 nm, respectively.

### Immunofluorescence

Rat corpus cavernosum tissues or CCSMCs were subjected to 30-min fixation in 3% methanol at 25 °C and subsequently rinsed thrice with PBS. Then, tissues or cells were incubated with 1% Triton X-100 at 25 °C for 5 min and sealed by 5% bovine serum albumin (BSA, Beyotime) at 37 °C for 1 h. Tissues or cells were incubated with anti-H3Cit (1: 200; #ab5103, Abcam, Cambridge, UK) and anti-MPO (1:100; #ab90810, Abcam) primary antibodies at 4 °C overnight, then Goat anti-Rabbit IgG H&L (Alexa Fluor^®^ 647) secondary antibody (1:500, #ab150115, Abcam). After 4',6-diamidino-2-phenylindole (DAPI; #C1005, Beyotime) staining at 25 °C for 1 h in darkness, the stained tissues or cells were photographed by a confocal imaging system (UltraVIEW VoX; Perkin Elmer, Waltham, MA, USA).

### Immunohistochemistry

Rat corpus cavernosum tissues were paraffinized, sectioned into 5 µm-slices, dewaxed, and rehydrated. To quench endogenous peroxidase activity, 3% H_2_O_2_ was added with the sections for 10 min. For antigen retrieval, the sections were immersed in citrate buffer (0.1 mol/L) for 10 min. After 1-h sealing with 5% BSA, the sections were incubated with anti-NLRP3 primary antibody overnight at 4 °C. Following three rounds of PBS washing, the sections were subjected to incubation with Goat anti-Rabbit IgG H&L (Alexa Fluor^®^ 488) secondary antibody (1: 10,000; #ab150077, Abcam). For visualization, 3,3'-diaminobenzidine (DAB; #P0202, Beyotime) was utilized. The confocal imaging system (UltraVIEW VoX; Perkin Elmer) was used to capture the images of sections.

### Cell counting kit-8 (CCK-8) assay

CCSMC viability was evaluated through CCK-8 assay. CCSMCs were seeded into 96-well plates (2 × 10^3^cell/well) and cultured at 37 °C with 5% CO_2_ for 24 h. Following 48-h incubation, CCK-8 solution (10 µL; #C0037, Beyotime, Shanghai, China) was added into per well for 2 h. Cell viability was assessed at 24 h and the OD at 450 nm was detected using a microplate reader (DR-3518G, Hiwell Diatek).

### Flow cytometry and observation of pyroptosis by microscopy

Flow cytometry was employed to assess CCSMC apoptosis. CCSMCs were suspended in 195 µL Annexin V-fluorescein isothiocyanate (FITC) binding buffer. Then, cells were stained with Annexin V-FITC (5μL) and propidium iodide (10 µL) (#C1062S, Beyotime) at 25 °C for 15 min and 10 min, respectively. A flow cytometer (CytoFLEX S; Beckman, Miami, FL, USA) was utilized for cell apoptosis assessment, and Cell Quest software (BD Biosciences, Franklin Lakes, NJ, USA) was for quantification.

To assess pyroptosis morphology, CCSMC cells were subjected to HG conditions for 24 h. The images were taken from four arbitrarily chosen areas using a microscope (DM3000, Leica, Germany).

### qRT-PCR

Isolation of total RNA from CCSMCs was realized using Trizol (#15596018, Thermo Fisher Scientific). To synthesize cDNA, reverse transcription was conducted via FastKing-RT SuperMix (#KR118-02, Tiangen, Beijing, China). qRT-PCR was executed on a CFX Connect RT-PCR detection system (Bio-Rad, CA, USA) through SYBR Green PCR Master Mix (#4364344; Thermo Fisher Scientific). Below is the reaction program applied: 95 °C, 3 min; 95 °C, 12 s; 62 °C, 40 s (40 cycles). The 2^−ΔΔCt^ method was employed to compute gene expression, standardized by glyceraldehyde-3-phosphate dehydrogenase (GAPDH) expression. The primers for this assay are exhibited in Table S1.

### Western blotting

Extraction of total protein from rat corpus cavernosum tissues or CCSMCs was conducted using radioimmunoprecipitation assay (RIPA) lysis buffer (#P0013B, Beyotime). Then, a bicinchoninic acid (BCA) kit (#PC0020, Solarbio) was employed for protein quantification. Proteins were isolated by 10% sodium dodecyl sulfate–polyacrylamide gel electrophoresis (SDS–PAGE; #P0015A, Beyotime) and migrated onto polyvinylidene difluoride (PVDF) membranes (#FFP24, Beyotime). Subsequently, the membranes were sealed with 5% nonfat milk (Beyotime) for 1 h, followed by primary antibody incubation at 4 °C overnight and Goat anti-Rabbit IgG H&L (HRP) secondary antibody (1: 1000, #A0208, Beyotime) incubation for 1 h. The membranes were washed for 3–5 min using enhanced chemiluminescence color developing solution (A solution and B solution are mixed 1:1) (#34579, Pierce, Rockford, IL, USA), and were packaged using a cling film. Next, the film was transferred to exposure cassette for exposure. During this process, the film was placed into developing solution for 2 min, and then was placed into fixing bath. Finally, ImageJ software was used for quantification, and GAPDH served as the internal reference. Primary antibodies used were as follows: anti-NLRP3 (1:1000; #AF2155, Beyotime), anti-GSDMD (1: 1000; #ab219800, Abcam), anti-GSDMD/GSDMD-N (1: 1000; #ab219800, Abcam), anti-pro-caspase-1/cleaved-caspase-1 (4 µg/mL; #ab286125, Abcam), anti-ASC (1: 1000; #ab307560, Abcam), anti-H3Cit (1: 2000; #ab5103, Abcam), and anti-GAPDH (1: 10,000; #ab181602, Abcam).

### Statistical analysis

Data were displayed as mean ± standard deviation. Statistical analyses were carried out using GraphPad 7.0 software (La Jolla, CA, USA). If the data showed a normal distribution, student’s t-test was used to evaluate the differences between two groups, and one-way ANOVA with Tukey’s test was applied for comparisons of multiple groups. If the data exhibited a non-normal distribution, the Mann–Whitney U test was used to analyze the differences between two groups, and the Kruskal–Wallis test followed by the Student–Newman–Keuls test was employed for comparisons of multiple groups. A *p* value below 0.05 was deemed significant difference.

### Supplementary Information

Supplementary Information 1.

Supplementary Information 2.

Supplementary Information 3.

Supplementary Information 4.

Supplementary Information 5.

Supplementary Table S1.

## Data Availability

The datasets generated during and/or analysed during the current study are available from the corresponding author on reasonable request.
