# Supplementary figures and images for: Neutrophil extracellular traps promote erectile dysfunction in rats with diabetes mellitus by enhancing NLRP3-mediated pyroptosis
Source: Sci Rep. 2024 Jul 16;14:16457. doi: 10.1038/s41598-024-67281-6 (PMC11252272; doi:10.1038/s41598-024-67281-6)

Figure 3B.

ASC


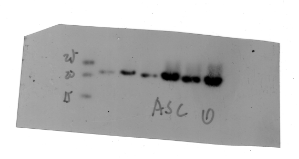


Caspase 1


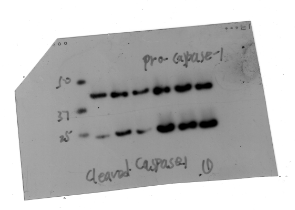


GSDMD


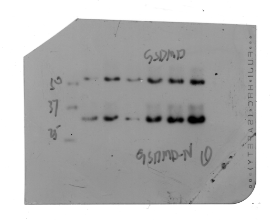


NLRP3


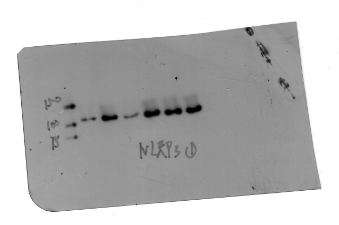


GAPDH


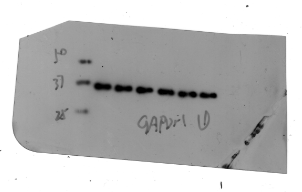

Supplement: Supplementary file 1 — Supplementary Information 1. [file 41598_2024_67281_MOESM1_ESM.docx]

Figure 5B

ASC


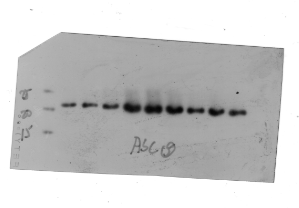


Caspase-1


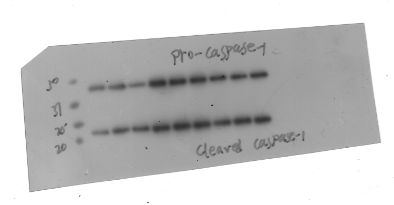


GSDMD


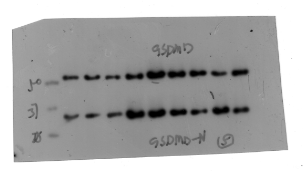


NLRP3


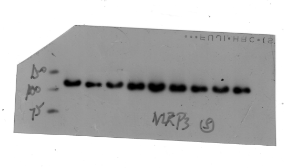


GAPDH


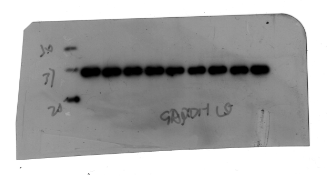

Supplement: Supplementary file 2 — Supplementary Information 2. [file 41598_2024_67281_MOESM2_ESM.docx]

Figure 6E

ASC


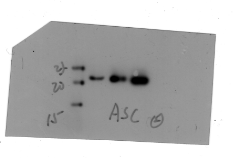


Caspase-1


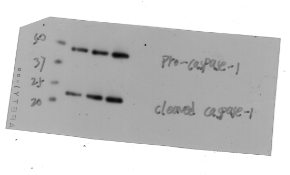


GSDMD


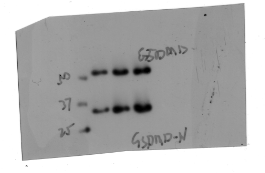


NLRP3


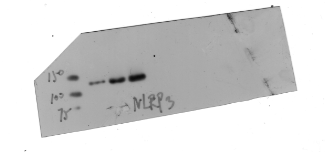


GAPDH


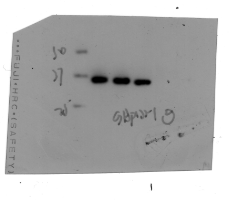

Supplement: Supplementary file 3 — Supplementary Information 3. [file 41598_2024_67281_MOESM3_ESM.docx]

Figure 7B

NLRP3


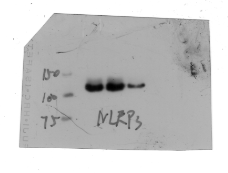


GAPDH


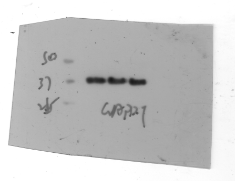


Figure 7D

NLRP3


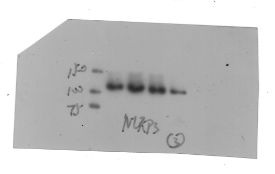


GAPDH


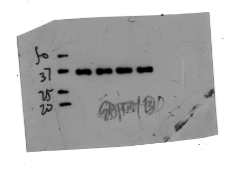


Figure 7E

H3Cit


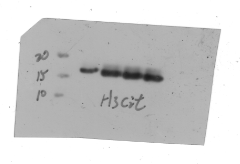


GAPDH


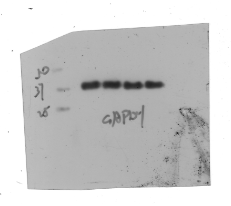

Supplement: Supplementary file 4 — Supplementary Information 4. [file 41598_2024_67281_MOESM4_ESM.docx]

Figure 8A

ASC


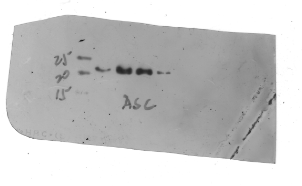


Caspase-1


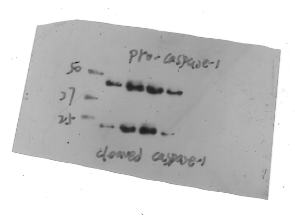


GSDMD


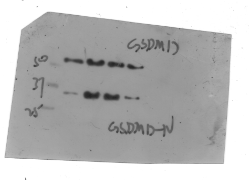


GAPDH


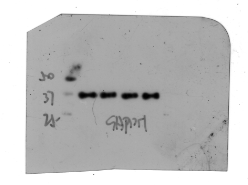

Supplement: Supplementary file 5 — Supplementary Information 5. [file 41598_2024_67281_MOESM5_ESM.docx]
