# Supplementary material for: Neutrophil extracellular traps promote erectile dysfunction in rats with diabetes mellitus by enhancing NLRP3-mediated pyroptosis
Source: Sci Rep. 2024 Jul 16;14:16457. doi: 10.1038/s41598-024-67281-6 (PMC11252272; doi:10.1038/s41598-024-67281-6)
Supplement: Supplementary file 6 — Supplementary Table S1. [file 41598_2024_67281_MOESM6_ESM.docx]

| Name | Primer sequence (5’ to 3’) |
| --- | --- |
| sh-NLRP3 (rat) | Sense strand: GGAAGCTCTTCAAAGACAA |
|  | Antisense strand: TTGTCTTTGAAGAGCTTCC |
| NLRP3 (rat) | Forward: ACGGCAAGTTCGAAAAAGGC |
|  | Reverse: AGACCTCGGCAGAAGCTAGA |
| GAPDH (rat) | Forward: GCGAGATCCCGCTAACATCA |
|  | Reverse: CTCGTGGTTCACACCCATCA |

**Table S1**. Primers used in this study.
